# Efficacy and Safety of Adalimumab in Noninfectious Uveitis: A Systematic Review and Meta-Analysis of Randomized Controlled Trials

**DOI:** 10.3389/fphar.2021.673984

**Published:** 2021-04-26

**Authors:** Biao Li, Haoran Li, Li Zhang, Yanlin Zheng

**Affiliations:** Department of Ophthalmology, Affiliated Hospital of Chengdu University of Traditional Chinese Medicine, Chengdu, China

**Keywords:** adalimumab, noninfectious uveitis, anti-TNF-α, treatment, meta-analysis

## Abstract

**Background:** Patients with noninfectious uveitis (NIU) are at risk of systemic side effects of long-term glucocorticoid therapy and uncontrolled inflammatory complications. In urgent need to identify more aggressive therapies, adalimumab (ADA) may be the right choice.

**Objectives:** To summarize the current evidence from randomized controlled trials (RCTs) regarding the efficacy and safety of ADA in the treatment of NIU.

**Methods:** We searched Pubmed, Embase, Web of Science, Cochrane Library databases, and Clinical Trials Registry for qualifying articles from their inception to November 19, 2020, with no language restriction. Randomized controlled trials comparing ADA with conventional routine treatment in noninfectious uveitis patients of any age, gender, or ethnicity were included. The primary outcome was the time to treatment failure (TF). The secondary outcomes were the change in best-corrected visual acuity (BCVA), change in the anterior chamber (AC) cell grade, change in vitreous haze (VH) grade, and adverse events (AEs).

**Main results:** The six studies comprised 605 participants in all, and the sample size of each study ranged from 16 to 225. The overall pooled results of the primary outcome (HR = 0.51; 95% CI, 0.41 to –0.63) showed that ADA nearly halved the risk of treatment failure compared to placebo for NIU patients. The pooled mean difference of change in BCVA was -0.05 (95% CI, −0.07 to −0.02). The pooled mean difference of change in AC cell grade and VH grade was −0.29 (95% CI, −0.62 to −0.05) and −0.21 (95% CI, −0.32 to −0.11), respectively. The incidence of AEs in the ADA group was numerically higher than that of AEs in the placebo group (2,237 events and 9.40 events per patient-year, equivalent to 1,257 events and 7.79 events per patient-year).

**Conclusion:** This meta-analysis of six RCTs further confirmed that ADA considerably lowered the risk of treatment failure or visual loss, and moderately reduced AC cell grades and VH grades with slightly more AEs, as compared to placebo. ADA is both effective and safe in treating NIU.

**Systematic Review Registration:** [https://clinicaltrials.gov], identifier [CRD42020217909].

## Introduction

Noninfectious uveitis (NIU) encompasses a heterogeneous collection of ocular disorders related to different etiologies, characterized by intraocular inflammation in the absence of infection (Airody et al., 2016; Dick et al., 2016). It is generally believed that noninfectious uveitis is an immune-mediated ocular inflammation frequently accompanied by systemic autoimmune diseases such as juvenile idiopathic arthritis, Behcet syndrome, or ankylosing spondylitis (Cordero-Coma and Sobrin, 2015; Schwartzman and Schwartzman, 2015). The mean prevalence of uveitis in Europe is 144.85 in 100,000 people, while NIU approximately accounts for 70% of uveitis (Llorenç et al., 2015). Simultaneously, the gross prevalence of NIU in American adults is roughly calculated to be 121/100,000 (Dick et al., 2016). It is estimated that the risk of blindness or low vision in patients with NIU is ten times higher than that in people without NIU (Durrani et al., 2004). NIU accounted for approximately 20% of legal blindness in developed countries, causing a massive burden to society (Nussenblatt, 2005; Wakefield and Chang, 2005; Jabs et al., 2013).

The treatment principle of NIU is to control intraocular inflammation, prevent relapses of inflammation, and reduce drug-related side effects. Currently, corticosteroids and immunosuppressants remain the mainstay of treatment drugs, which sometimes fail to control inflammation and frequently cause well-known ocular and systemic adverse effects (Sen et al., 2014; Miloslavsky et al., 2017; Niederer et al., 2017; Suhler et al., 2017). Therefore, it is urgent to identify more effective and safer therapies that target specific immune response mediators to achieve and maintain inflammation remission. Moreover, adalimumab (ADA) may be the one (Suhler et al., 2013; Levy-Clarke et al., 2014).

Adalimumab (Humira®; AbbVie Inc.) is a full-length human monoclonal antibody that uniquely targets TNF-α and counteracts its biological activity (Burmester et al., 2013; Airody et al., 2016; Balevic and Rabinovich 2016). Currently, ADA is the only biologic that has been proven effective to NIU by randomized-control, double-blind phase Ⅲ studies (Jaffe et al., 2016; Nguyen et al., 2016). Consequently, the United States, European countries, Japan, and China have successively approved ADA to treat NIU. Although it received approval in these countries, ADA has not yet been widely used worldwide.

The most recent systematic review on the ADA for NIU dates back to 2018 (Ming et al., 2018), while several relevant articles have been published after that. Moreover, all the existing systematic reviews on this topic are mainly based on observational studies and rarely include randomized controlled trials (RCTs) which lead to their low quality of evidence. So we herein conducted a systematic review with meta-analysis to synthesize the currently accessible evidence from RCTs to assess ADA’s efficacy and safety in NIU.

## Article Types

A Systematic Review and Meta-Analysis of RCTs.

## Manuscript Formatting

### Methods

This meta-analysis was conducted following the Preferred Reporting Items for Systematic Review and Meta-analysis statements (Shamseer et al., 2015), with the protocol registered in the Prospero database (CRD42020217909).

#### Study Design and Interventions

The main inclusion criteria are as follows: 1) randomized controlled trials comparing ADA with conventional routine treatment (such as local and systemic corticosteroids, immunosuppressants) in patients of any age, gender, or ethnicity with a diagnosis of NIU; 2) the mean follow-up duration was more than three months; 3) sample size greater than 10; 4) AC cell grade and VH grade were evaluated by Standardization of Uveitis Nomenclature (SUN) criteria (Jabs, Nussenblatt, and Rosenbaum, 2005). We excluded studies that met any of the following conditions: 1) duplicate reports on the same study; 2) inadequate data or information; 3) control group was not placebo.

#### Data Sources and Search Strategy

Pubmed, Embase, Web of Science, Cochrane Library, and Clinical Trials Registry were searched for relevant literature from their inception to November 19, 2020, regardless of language. If necessary, the researchers were contacted for more data. The search was limited to abstract/keyword/title fields. The search terms included uveitis, iridocyclitis, retinitis, retinal vasculitis, panuveitis, uveit*, adalimumab, ADA, Humira, TNF, TNF-a, anti-tumor necrosis factor-alpha, randomized controlled trial, and clinical trial. The Boolean operators appropriately connected these keywords. Study design types were restricted to randomized controlled trials (RCTs).

#### Study Selection and Exclusion Processes

According to the inclusion criteria, two reviewers (Biao Li and Haoran Li) independently assessed the relevant studies for eligibility. Any disagreements were resolved through discussion among ourselves.

#### Outcomes Assessment

The primary outcome was the time to treatment failure (TTF), a rigorous composite outcome composed of four components (new ocular inflammatory lesions, BCVA, AC cell grade, and VH grade). “Treatment failure” was defined by the presence of one or more of the following factors: 1. new active, inflammatory lesions relative to baseline; 2. a two-step increase in anterior chamber cell or vitreous haze grade; 3. a worsening of best-corrected visual acuity by 15 or more letters on the Early Treatment Diabetic Retinopathy Study chart, relative to the best state previously achieved, in at least one eye; 4. sustained non-improvement with entry grade of ≥3; 5. use of concomitant medications not allowed; and 6. intermittent or continuous suspension of study treatment (adalimumab or placebo) for a cumulative period of longer than four weeks. The secondary efficacy outcomes included change in BCVA (logMAR), change in AC cell grade, and VH grade (according to SUN). The safety outcome was the number and the rate of adverse events (per patient-years).

#### Data Extraction

Two authors (Biao Li and Haoran Li) extracted the data from the included publications into standard forms independently and cross-checked them to ensure accuracy. Differences were settled by discussion and transferred to a third author if needed. The information captured included: first author’s last name, published date, number of patients in each group, demographic data, follow-up time, and definitions of endpoints. If the same registered trial data appeared in multiple articles, the article with the latest or most comprehensive data was included.

#### Data Analysis

Two reviewers (Biao Li and Li Zhang) independently evaluated the quality of the included studies using the recommended Cochrane Collaboration tool for assessing the risk of bias, which consists of seven types of risks of bias: random sequence generation; incomplete outcome data; allocation concealment; selective reporting; blinding of participants and personnel; blinding of outcome assessment; for-profit bias. Disagreements were settled by discussion and transferred to a third author if needed. We conduct our statistical analysis using review manager version 5.3 software according to the intention to treat analysis method. The evaluation of outcomes was done per eye, except TF and AEs, mainly pooled per patient. For continuous endpoints such as BCVA, AC cell grade, and VH grade, we preferentially retrieved mean differences with 95% CI. When meta-analysis was not proper for certain types of data we narratively summarize the relevant results.

The heterogeneity of the pooled results was assessed by Cochrane’s Q test and Higgins’ I^2^. If apparent heterogeneity existed (*p*<0.1 or I^2^ > 50%), the pooled results were estimated using the random-effects model. Alternatively, the fixed-effects model was adopted. Besides, we deleted each study to assess each study’s impact on the overall risk estimate to examine the results’ robustness. Subgroup analysis was initially planned according to the type of uveitis, study location, follow-up time duration, and participants’ age. Unfortunately, we do not have enough sample size to perform these analyses.

We originally planned to examine the publication bias of included studies by funnel plots and Egger’s test. Unfortunately, we lack enough studies to conduct these analyses.

#### Confidence in Cumulative Evidence

The GRADE system was used to access the evidence’s quality of every efficacy outcomes. GRADE system scored the evidence of each outcome in five aspects: limitations of the study design and execution, inconsistency, indirectness, imprecision of results, and publication bias. Accordingly, we classify ADA treatment’s recommendation level as very low, low, medium, or high.

### Results

#### Study Selection

**FIGURE 1:**
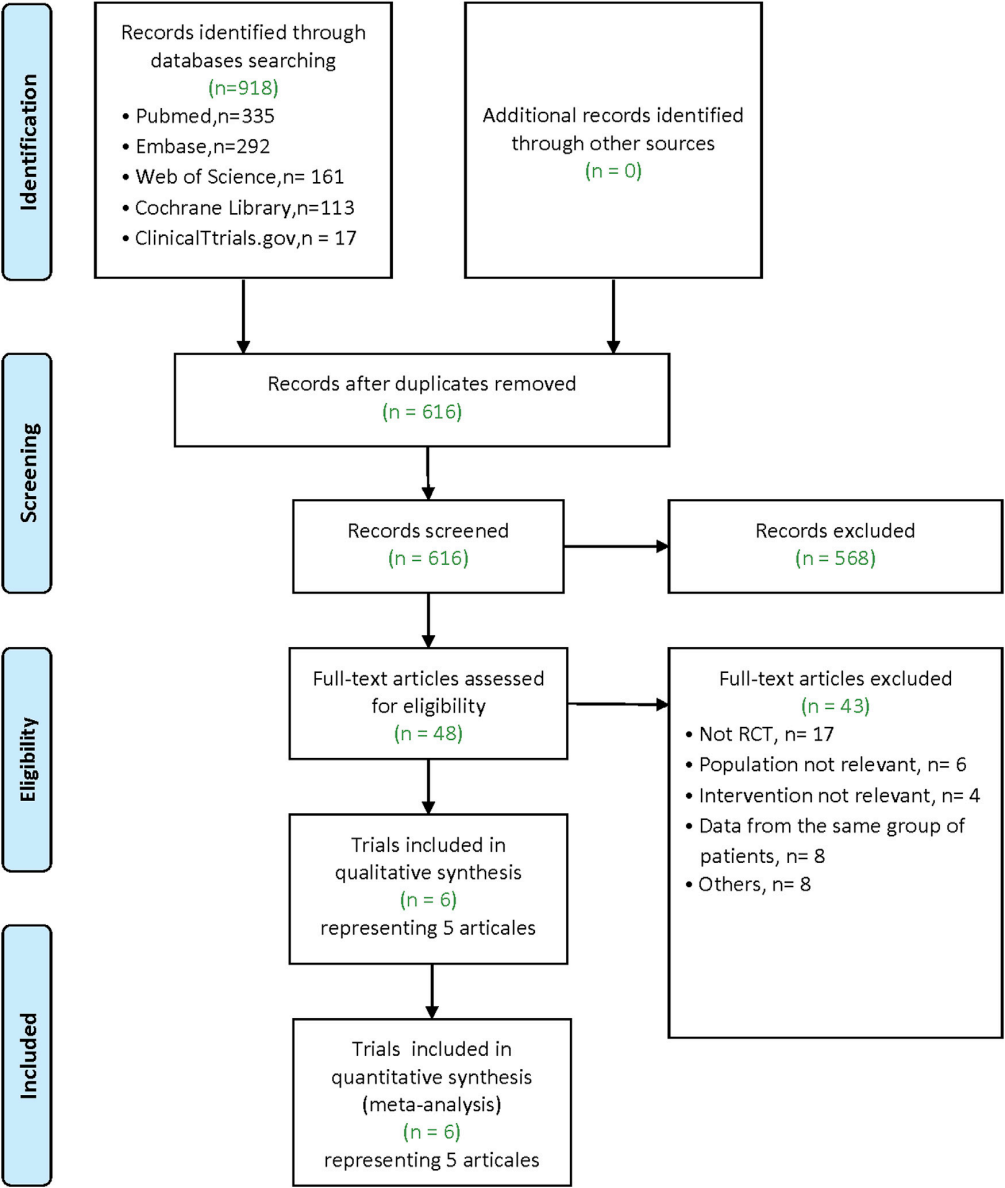
Flow diagram.

Our literature search yielded 918 articles (Pubmed: 335; Embase: 292; Web of Science: 161; Cochrane Library: 113; ClinicalTtrials.gov: 17). After removing duplicates, 616 articles remain. Of these, 568 were excluded after screening for the titles and abstracts. After the full-text examination of the remaining 48 articles, five articles (6 trials, there was one article containing the data of two RCTs) met inclusion criteria for our meta-analysis (Figure 1).

#### Study Characteristic

All six studies used intention-to-treat analysis to evaluate efficacy and safety outcomes. Six hundred thirty-six patients were enrolled in six studies, including 318 in the ADA group and 287 in the control group. The sample size ranged from 16 to 225. Overall, 61.23% (368/601) of patients were female, with the sex distribution favored females in each of the six studies. The mean age varied from 8.90 to 50.90 years old, and the mean uveitis duration was between 43.75 and 94.56 months. Table 1 summarises the characteristics of the six selected RCTs.

**TABLE 1:** Characteristics of the included RCTs.

	Study, study dates, setting, type of study, registration number	Sample size (ADA/placebo), mean age, % of females, type of uveitis, uveitis duration	Population: diagnosis	Intervention and comparator	Outcomes
VISUAL I main	• August 2010–August 2014 • 67 sites, 18 countries • Multicenter, double-masked, randomized Placebo-controlled Phase 3 trial • NCT 01138657	• *n* = 217 (110/107), age 42.65 (14.89)years 57% female • Active uveitis • Intermediate 22%, posterior 33%, pan 45% • Bilateral 91%, unilateral 9% • 45.53 (62.53)months	• Idiopathic 37% • Sarcoidosis 8% • Behçet’s disease 7% • VKH 12% • Birdshot chorioretinopathy 20% • Multifocal choroiditis and panuveitis 5% • Other 10%	• Intervention: subcutaneous ADA, 80 mg loading dose followed by 40 mg dose eow • comparator: placebo • prednisone burst for all at week 0, tapering to 0 by week < 15	• Primary outcome: TTF (worsening of one or more of AC grade, VH grade, BCVA, or new inflammatory lesions) at/after week 6, one or more eyes • Secondary outcomes: BCVA, change in VH or AC grade, % change in CRT, time to MO, change in VFQ-25 score, AEs
VISUAL II main	• August 2010–May 2015 • 72 sites, 22 countries • Multicenter, double-masked, randomized placebo-controlled, phase 3 trial	• *n* = 225 (114/111), age 42.56 (13.43) years 57% female • Inactive uveitis • Intermediate 21%, posterior 33%, pan 46% • Bilateral 96%, unilateral 4% • 61.17 (65.97)months	• Idiopathic 31% l • Sarcoidosis 14% • Behçet’s disease 7% • VKH 23% • birdshot chorioretinopathy 13% • multifocal choroiditis and Panuveitis 3% • other 9%	• Intervention: subcutaneous ADA, 80 mg loading dose followed by 40 mg dose eow • Comparator: placebo • Prednisone burst for all at week 2, tapering to 0 by week 15	• Primary outcome: TTF (worsening of one or more of AC grade, VH grade, BCVA, or new inflammatory lesions) at after week 2, one or more eyes • Secondary outcomes: BCVA, change in VH or AC grade, % change in CRT, time to MO, change in VFQ-25 score, AEs
VISUAL I Japan	• August 2010–August 2014 • 7 sites in Japan • Multicenter, double-masked, randomized Placebo-controlled Phase 3 trial • NCT 01138657	• *n* = 16 (8/8), age 50.9 (14.72) years, 59% female • Active uveitis • Intermediate 6%, posterior 13%, pan 81% • Bilateral 87.5% Unilateral 12.5% • 57.15 (75.70)months	• Idiopathic 44% • Sarcoidosis 38% • Behçet’s disease 12% • VKH 6%	• Intervention: subcutaneous ADA, 80 mg loading dose followed by 40 mg dose eow • Comparator: Placebo • Prednisone burst for all at week 0, tapering to 0 by week 15	• Primary outcome: TTF (worsening of one or more of AC grade, VH grade BCVA, or new inflammatory lesions) at/after week 6, one or more eyes • Secondary outcomes: BCVA, change in VH or AC grade, % changein CRT, time to MO, change in VFQ-25 score, AEs
VISUAL II Japan	• August 2010–May 2015 • 7 sites in Japan • Multicenter, double-masked, randomized Placebo-controlled Phase 3 trial • NCT 01124838	• *n* = 32 (16/16), age 46.8 (12.49) years, 59% female • Active uveitis • Intermediate 0%, posterior 9%, pan 91% • Bilateral 91%, unilateral 9% • 43.75 (38.13)months	• Idiopathic 25% • Sarcoidosis 31% • Behçet’s disease 3% • VKH 38% • Other 3%	• Intervention: subcutaneous ADA, 80 mg loading dose followed by 40 mg dose eow • Comparator: placebo • Prednisone burst for all at week 2, tapering to 0 by week 15	• Primary outcome: TTF (worsening of one or more of AC grade, VH grade, BCVA, or new inflammatory lesions) at after week 2, one or more eyes • Secondary outcomes: BCVA, change in VH or AC grade, % change in CRT, time to MO, change in VFQ-25 score, AEs
Mackensen 2018	• May 2007–August 2012 • 2 centers • Randomized, prospective, controlled Two-center clinical trial • NCT 00348153	• *n* = 25 (10/15), age 36 years, 60% female • Active uveitis • Anterior 60%, posterior + pan 40%- • 94.56 months	• JIA: 8% • Spondyloarthritis:16% • GPA/Behçet’s/sarcoidosis: 20% • HLA-B27B: 24%	• Intervention: ADA (40 mg subcutaneous injection eow) • Comparator: blank • Both arms continued previous immunosuppressive therapy and received a corticosteroid bolus of 1 mg/kg bw, with a fixed standardized tapering scheme	• Primary outcome: change in visual acuity • Secondary outcomes: extent of macular edema, intraocular inflammatory Activity (SUN), the number of treatment arm switchers, the cumulative systemic corticosteroid dose, AEs
Ramanan 2019	• October 2011–June 2015 • 14 centers in the United Kingdom • Randomized, parallel-group, double-blind Placebo-controlled Multicenter clinical trial • ISRCTN 10065623	• *n* = 90 (60/30), age 8.90 (3.88) years, 78% female • Active uveitis • Bilateral 28%, unilateral 72% • 63.96 (42.36)months	JIA-associated uveitis 100%	• Intervention: ADA (20 mg/0.8 ml for patients weighing <30 kg or 40 mg/0.8 ml for patients weighing ≥30 kg by subcutaneous injection eow) • Comparator: placebo • All participants received a stable dose of MTX	• Primary outcome: TTF (multicomponent score as defined by set criteria based on the SUN criteria) • Secondary outcomes: number of participants failing treatment, BCVA use of corticosteroids, safety, tolerability, compliance

ADA, adalimumab; eow, every other week; AC, anterior chamber; AE, adverse event; CRT, central retinal thickness; JIA, juvenile idiopathic arthritis; LFP laser flare photometry; MTX, methotrexate; SL; SUN, standardization of uveitis nomenclature; TTF, time to treatment failure; VH, vitreous haze.

#### Quality Assessment

We judged for-profit bias in all six studies as high risk since five of the included studies were sponsored by pharmaceutical companies (AbbVie), while the remaining one studies had participants who received remuneration such as speaker’s fees from AbbVie.

**FIGURE 2:**
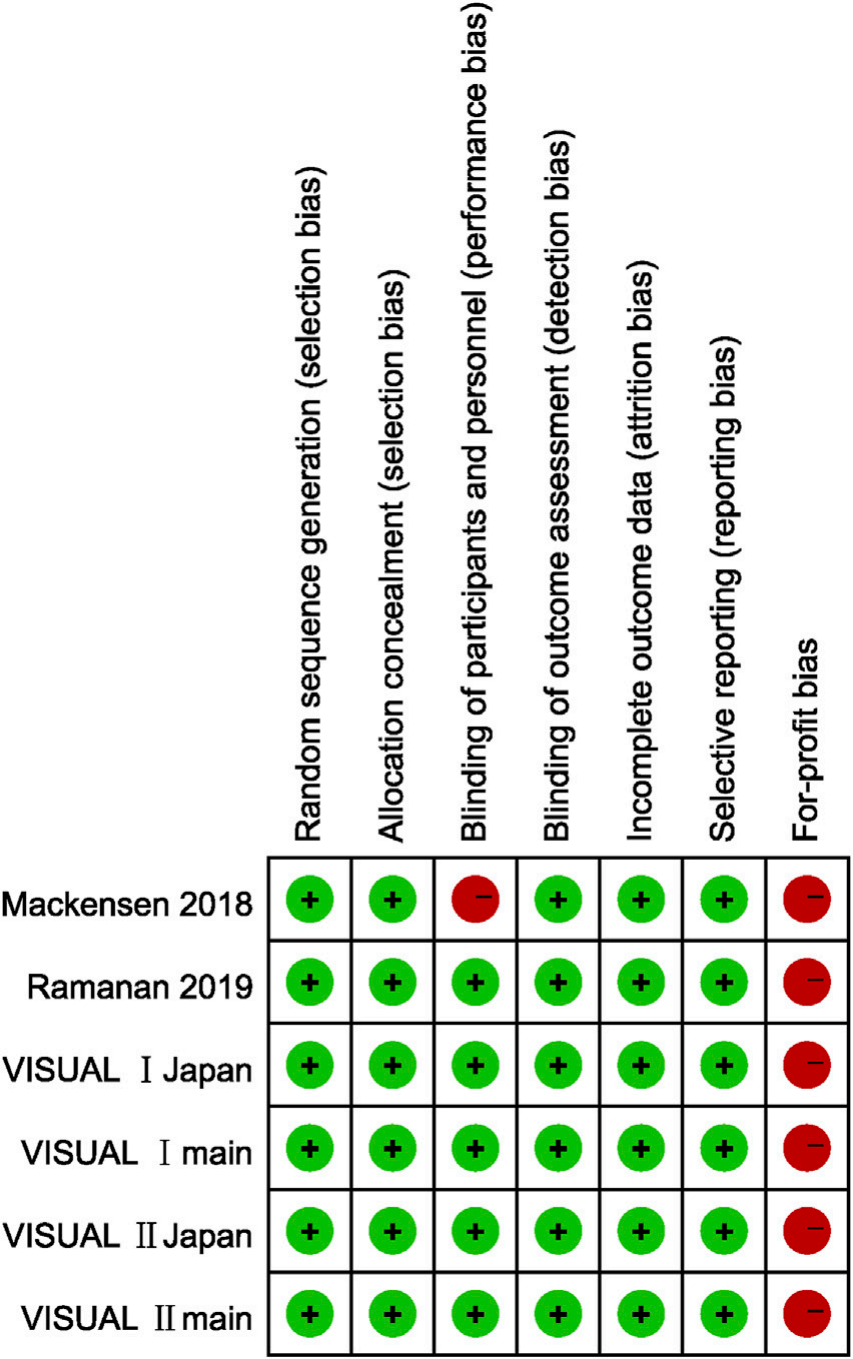
Risk of bias summary.

Since only outcome assessors were blinded in Mackensen’s study, the risk of performance bias was considered high, and the detection bias was assessed as low risk. In the other five studies, the risk regarding blinding were all considered low risk since the methods of blinding patients, doctors, and outcome assessors were described in detail. The remaining four types of risk of bias were all considered low risk in six studies because the corresponding evidence can be found in the article. As prospective trial registrations were accomplished in all studies and their prespecified outcomes were reported, selective reporting was considered low risk. Figure 2 demonstrates the risk of bias in each study.

#### Synthesis of Results

The pooled results included the time to treatment failure, change in AC cell grade, change in VH grade, and change in BCVA (logMAR). Table 2 demonstrates the relevant results of the including studies.

**TABLE 2:** Summary of the results of individual studies.

	Time to treatment failure, HR	BCVA (change) (logMAR), MD	AC cell grade (change), MD	VH grade (change), MD
VISUAL Ⅰ main	0.50 (0.36, 0.70)	−0.07 (−0.11, −0.02)	−0.29 (−0.51, −0.07)	−0.27 (−0.43, −0.11)
VISUAL Ⅱ main	0.57 (0.39, 0.84)	−0.04 (−0.08, 0.01)	−0.14 (−0.37, 0.08)	−0.13 (−0.28, 0.01)
VISUAL Ⅰ Japan	1.20 (0.41, 3.54)	0.04 (−0.22, 0.31)	0.22 (−0.17, 0.61)	−0.41 (−1.15, 0.34)
VISUAL Ⅱ Japan	0.45 (0.20, 1.03)	−0.08 (−0.20, 0.04)	−0.22 (−0.90, 0.46)	−0.45 (−0.98, 0.07)
Mackensen 2018	NA	NA	−0.43 (−1.05, 0.18)	−0.54 (−1.22, 0.14)
Ramanan 2019	0.25 (0.12, 0.51)	−0.02 (−0.07, 0.02)	−0.79 (−0.96, −0.63)	NA

NA, not available; MD, mean difference; SD, standard deviation; HR, hazard ratio; BCVA, best-corrected visual acuity; AC, anterior chamber; VH, vitreous haze; logMAR, logarithm of the minimum angle of resolution.

##### Time to Treatment Failure

**FIGURE 3:**
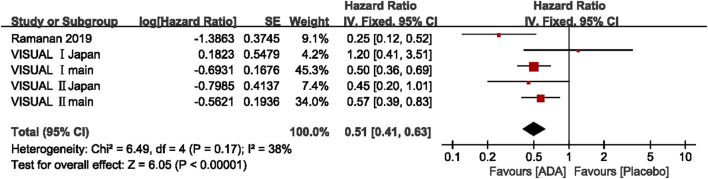
Forest plot of time to treatment failure.

The hazard ratio (HR) to treatment failure was reported in five studies. All studies reported the HRs ranging from 0.25 to 0.57, except for the VISUAL Ⅰ  Japan (HR = 1.2, 95% CI, 0.41–3.51). The pooled results of all the five studies (HR = 0.51; 95% CI, 0.41–0.63) showed that ADA nearly halved the risk of treatment failure compared to placebo. Heterogeneity was not significant by the Q statistic (6.49 on 4 df, *p* = 0.17) and by *I* ^2^ (38%). (Figure 3)

##### Change in BCVA (logMAR)

**FIGURE 4:**
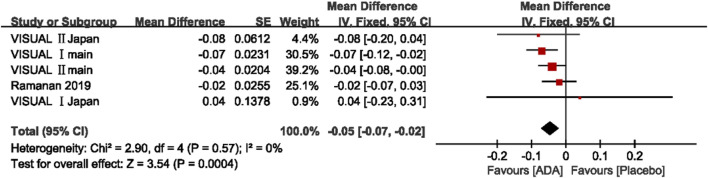
Forest plot of BCVA.

A total of five studies involving 856 patients presented the change in BCVA (logMAR) after the intervention. The mean difference of the change in BCVA ranged between −0.08 and 0.04 (*p* <0.05). The pooled estimate mean difference favored patients who received ADA (MD = −0.05, 95% CI, −0.07 to −0.02, *p* = 0.0004). The Q statistic (2.91 on 5 df, *p* = 0.71) and I^2^ (0%) indicated low heterogeneity in the pooled studies. (Figure 4)

##### Change in AC Cell Grade

Of the six studies evaluating change in AC cell grade, five studies found that the ADA group’s AC cell grade improved significantly more than the placebo group, while one trial reported the opposite result. The mean difference was ranging from −0.79 to 0.22 (*p* <0.05). The pooled mean difference was −0.29 (95% CI, −0.62 to 0.05), not showing a significant difference between ADA and placebo groups. Both the Q statistic (38.58 on 5 df, *p* < 0.01) and by *I* ^2^ (87%) demonstrated significant heterogeneity, which indicated that a random-effects model was preferable.

Additionally, we excluded each study's estimates to examine each study’s influence on the overall results. After excluding VISUAL Ⅰ Japan, the overall results have changed a lot that the diamond marker does not intersect with 0, showing that the AC cell grade was significantly better in the ADA group than the placebo group (MD = −0.39, 95% CI: −0.72, −0.06).

**FIGURE 5:**
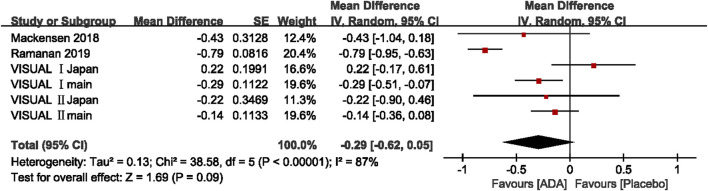
Forest plot of AC cell grade.

In VISUAL I Japan, AC cell grade was numerically higher in the ADA group (MD = 0.22; 95% CI, −0.17, −0.61) since one patient in the placebo group did not experience treatment failure from the beginning to end. Given the small sample size, this patient strongly impacted all the outcomes including AC cell grade. (Figure 5)

##### Change in VH Grade

**FIGURE 6:**
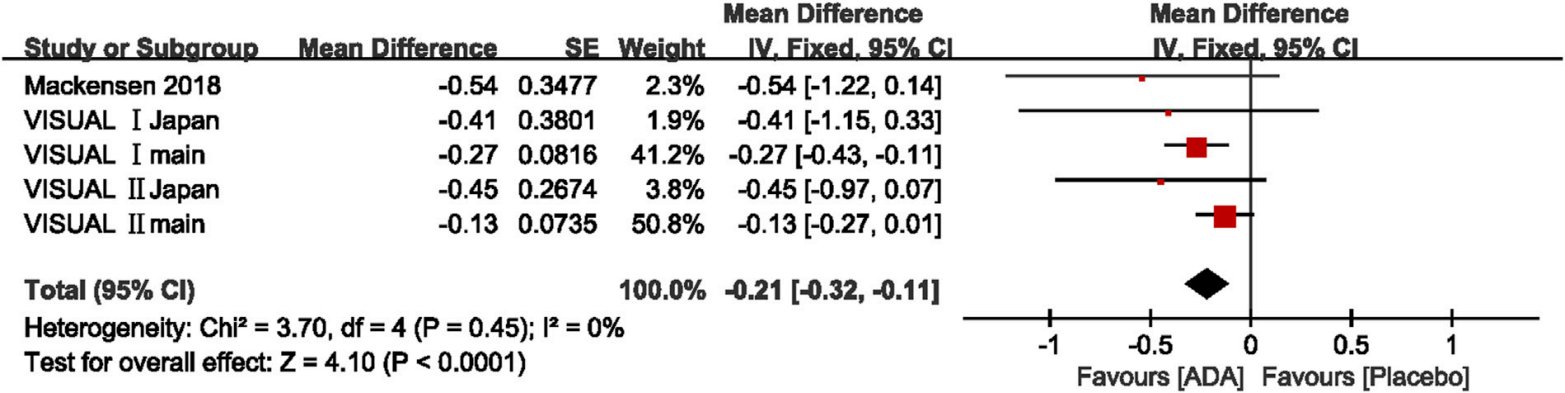
Forest plot of VH grade.

Five studies reported Change in VH grade, and the mean difference was ranging from −0.54 to −0.13 (*p* <0.05). The pooled mean difference in VH grade change was −0.21, with a 95% CI (−0.32, −0.11), suggesting that the VH grade improved significantly more in the ADA group than the control group. Heterogeneity was low by the Q statistic (3.70 on 4 df, *p* = 0.45) and by I^2^ (0%). After removing any one study, the pooled results did not change significantly, and the estimates in each case were well within the confidence range of the overall estimate. (Figure 6)

##### Safety

All six RCTs have safety information with 601 patients. A total of 3,494 AEs occurred during 406.5 patient-years, and the overall incidence of AEs was 8.60 events per patient-year. There were 2,237 AEs in the ADA group, and the overall incidence was 9.40 events per patient-year, numerically higher than those of the 1,257 total events and 7.79 events per patient-year in the placebo group. Table 3 shows the results of the safety. Six studies reported a total of 59 serious adverse events, of which 66% (39) occurred in the ADA group, indicating that the risk of serious adverse events in the ADA group was twice that of the placebo group.

**TABLE 3:** summary of safety result.

		ADA				Placebo			
AE summery	Sample size (ADA/placebo)	AEs, no. of events	AEs, events/patient-years	SAEs, no. of events	SAEs, events/patient-years	AEs, no. of events	AEs, events/patient-years	SAEs, no. of events	SAEs, events/patient-years
VISUAL 1 main	217 (110/107)	657	10.524	18	0.288	430	9.717	6	0.136
VISUAL 2 main	225 (114/111)	831	8.790	13	0.138	642	9.050	10	0.141
VISUAL 1 Japan	16 (8/8)	28	12.101	1	0.431	25	7.962	0	0
VISUAL 2 Japan	32 (16/16)	48	6.743	1	0.140	16	7.344	1	0.459
Mackensen 2018	25 (10/15)	54	7.665	1	0.142	30	5.475	0	0
Ramanan 2019	90 (60/30)	619	10.600	5	0.086	114	7.210	3	0.190

ADA, adalimumab; AE, adverse event; SAE, serious adverse event.

The most common AEs were injection-site reactions and allergic reactions. The AEs reported in six studies were similar to those reported in previous studies and no new AEs occurred.

#### Risk of Bias Across Studies

It cannot be performed due to the small sample size.

### Discussion

#### Summary of Main Findings

This meta-analysis of six RCTs including 605 patients systematically reviewed ADA’s efficacy and safety in NIU. The results show that ADA almost halved the risk of NIU patients’ treatment failure by significantly improving BCVA and reducing the AC cell grade and VH grade. The incidence of ADA-related AEs was generally low, and the safety profile was similar to other reports in previous studies. Significant differences favoring ADA over placebo was seen for two secondary endpoints (change in BCVA and VH grade). Outcomes regarding AC cell grade in the ADA group were numerically superior to that in the placebo group.

#### GRADE

According to the GRADE, the certainty of the evidence concerning four efficacy outcomes were all judged as moderate. Table 4 shows the summary of GRADE’s. Moderate-quality evidence shows that ADA considerably lowered the risk of treatment failure or visual impairment, moderately reduced AC cell grades and VH grades in NIU.

**TABLE 4:** GRADE’s summary of finding.

Adalimumab compared to Placebo for non-infectious uveitis						
Patient or population: non-infectious uveitis						
Setting:						
Intervention: Adalimumab						
Comparison: Placebo						

Outcomes No of participants (studies)	Relative effect (95% CI)	Anticipated absolute effects (95% CI) Difference			Certainty	What happens
		
Time to treatment failure	HR 0.51 (0.41 to 0.63) [Time to treatment failure]				⊕⊕⊕○ MODERATE	—
No of participants: 667 (5 RCTs)						
Change in BCVA (logMAR)	–		–	MD 0.05 lower (0.07 lower to 0.02 lower)	⊕⊕⊕○ MODERATE	—
№ of participants: 580 (5 RCTs)						
Change in AC cell grade	–		–	MD 0.29 lower (0.62 lower to 0.05 higher)	⊕⊕⊕○ MODERATE	—
№ of participants: 592 (6 RCTs)						
Change in VH grade	–		–	MD 0.21 lower (0.32 lower to 0.11 lower)	⊕⊕⊕○ MODERATE	—
№ of participants: 502 (5 RCTs)						

*The risk in the intervention group (and its 95% confidence interval) is based on the assumed risk in the comparison group and the relative effect of the intervention (and its 95% CI). CI: Confidence interval; HR: Hazard Ratio; MD: Mean difference

GRADE Working Group grades of evidence

High certainty: We are very confident that the true effect lies close to that of the estimate of the effect

Moderate certainty: We are moderately confident in the effect estimate: The true effect is likely to be close to the estimate of the effect, but there is a possibility that it is substantially different

Low certainty: Our confidence in the effect estimate is limited: The true effect may be substantially different from the estimate of the effect

Very low certainty: We have very little confidence in the effect estimate: The true effect is likely to be substantially different from the estimate of effect

#### Comparison to Prior Reviews

The efficacy and safety results mentioned above were consistent with those of a meta-analysis by Shuai Ming et al. Their review included 20 non-RCTs and three RCTs. In contrast, our study included six RCTs and excluded non-RCTs, leading to a higher quality of evidence.

A similar article entitled “Anti-TNF Drugs for Chronic Uveitis in Adults—A Systematic Review and Meta-Analysis of Randomized Controlled Trials” has been published by Leal et al. on May 24, 2019. Leal’s study included three RCTs concerning two drugs: adalimumab and etanercept, with a sample size of 458. In comparison, our meta-analysis included six studies related to one single drug (ADA) with a sample size of 605. Moreover, the efficacy analysis was not conducted in Leal's study because of the significant heterogeneity between interventions. Our study pooled the six studies for efficacy analysis using uniform outcome measures such as BCVA, change in AC cell grade, and VH grade (SUN). This review is the first meta-analysis including only RCTs to summarize the current evidence regarding ADA’s efficacy and safety in NIU, with strengths in the relatively higher quality of evidence and multicomponent primary endpoint.

#### Limitations

The principal limitation is that high heterogeneity was observed in our analysis. Differences in types of uveitis, patient’s age, concomitant medications, and outcome measures may contribute to the heterogeneity. It is important to note that we did not restrict participants’ age, so we recruited both adult and pediatric patients in this meta-analysis. In adults, ADA’s main indications in NIU are intermediate, posterior forms of uveitis and panuveitis. In children with uveitis, ADA’s main indication is JIA-associated uveitis and idiopathic uveitis, which is mainly anterior uveitis. We originally planned to conduct subgroup analysis in this article based on the location and type of uveitis, but due to the small sample size and the inability to extract relevant data from some studies, we gave up the subgroup analysis. In the future, more studies on the treatment of single uveitic disease with ADA are needed.

Besides, it is well known that RCT is not an ideal type of study to identify safety results because the relatively small sample size and short follow-up time make it challenging to identify rare adverse events. Therefore, the RCTs included in this review are not sufficient to study AEs thoroughly.

A further limitation was that the four VISUAL trials were sponsored by one pharmaceutical company (AbbVie) and the remaining two studies had participants who received remuneration such as the speaker’s fees from AbbVie, which may seriously affect the results.

#### Implication

In the future, independent non-company sponsored RCTs are needed to further provide more objective and robust evidence. Besides, it is necessary to further compare ADA with conventional immunosuppressors regarding efficacy and safety in NIU.

## Conclusion

This meta-analysis of six RCTs comparing ADA with placebo for NIU further confirmed that ADA considerably lowered the risk of treatment failure or visual impairment, and moderately reduced AC cell grades and VH grades with slightly more AEs.

## Data Availability

The original contributions presented in the study are included in the article/Supplementary Material; further inquiries can be directed to the corresponding author/s.
